# Rabies Diagnosis for Developing Countries

**DOI:** 10.1371/journal.pntd.0000206

**Published:** 2008-03-26

**Authors:** Salome Dürr, Service Naïssengar, Rolande Mindekem, Colette Diguimbye, Michael Niezgoda, Ivan Kuzmin, Charles E. Rupprecht, Jakob Zinsstag

**Affiliations:** 1 Swiss Tropical Institute, Basel, Switzerland; 2 Laboratoire de Recherches Vétérinaires et Zootechniques de Farcha, N'Djaména, Tchad; 3 Centre de Support en Santé International, N'Djaména, Tchad; 4 Centers for Disease Control and Prevention, Atlanta, Georgia, United States of America; University of Edinburgh, United Kingdom

## Abstract

**Background:**

Canine rabies is a neglected disease causing 55,000 human deaths worldwide per year, and 99% of all cases are transmitted by dog bites. In N'Djaména, the capital of Chad, rabies is endemic with an incidence of 1.71/1,000 dogs (95% C.I. 1.45–1.98). The gold standard of rabies diagnosis is the direct immunofluorescent antibody (DFA) test, requiring a fluorescent microscope. The Centers for Disease Control and Prevention (CDC, Atlanta, United States of America) developed a histochemical test using low-cost light microscopy, the direct rapid immunohistochemical test (dRIT).

**Methodology/Principal Findings:**

We evaluated the dRIT in the Chadian National Veterinary Laboratory in N'Djaména by testing 35 fresh samples parallel with both the DFA and dRIT. Additional retests (*n* = 68 in Chad, *n* = 74 at CDC) by DFA and dRIT of stored samples enhanced the power of the evaluation. All samples were from dogs, cats, and in one case from a bat. The dRIT performed very well compared to DFA. We found a 100% agreement of the dRIT and DFA in fresh samples (*n* = 35). Results of retesting at CDC and in Chad depended on the condition of samples. When the sample was in good condition (fresh brain tissue), we found simple Cohen's kappa coefficient related to the DFA diagnostic results in fresh tissue of 0.87 (95% C.I. 0.63–1) up to 1. For poor quality samples, the kappa values were between 0.13 (95% C.I. −0.15–0.40) and 0.48 (95% C.I. 0.14–0.82). For samples stored in glycerol, dRIT results were more likely to agree with DFA testing in fresh samples than the DFA retesting.

**Conclusion/Significance:**

The dRIT is as reliable a diagnostic method as the gold standard (DFA) for fresh samples. It has an advantage of requiring only light microscopy, which is 10 times less expensive than a fluorescence microscope. Reduced cost suggests high potential for making rabies diagnosis available in other cities and rural areas of Africa for large populations for which a capacity for diagnosis will contribute to rabies control.

## Author Summary

A new diagnostic test for rabies in animals was evaluated in N'Djaména, capital of Chad. The test is based on a direct immuno-histochemical detection of rabies virus in brain tissue (dRIT) visible by normal light microscopy. Rabies detection by dRIT light microscopy is 10 times less expensive than fluorescence microscopy required for the current gold standard of rabies diagnosis. The test showed ideal results in fresh samples with 100% agreement with the gold standard and confirms the results of a first study in Tanzania. Thus, it has a significant potential for diagnosing rabies in low-income countries, and under field conditions where rabies diagnosis is unavailable for the moment. This new test opens up a great potential to train technical staff and to establish rabies diagnosis without delay in low-income countries with urban rabies.

## Introduction

Canine rabies is an endemic disease in most developing countries. Worldwide, approximately 55,000 (90% CI: 24,500–90,800) human deaths occur per year [1]. In N'Djaména, the capital of Chad, Kayali et al found a rabies incidence in the dog population of 1.4/1000 (95% CI: 1.2–1.7) among unvaccinated dogs in 2001 [2]. In more than 99% of all human cases, the virus is transmitted by dog bite [3],[4]. Administration of post exposure prophylaxis (PEP) immediately after the animal bite is essential to prevent productive infection, which is essentially fatal. Full PEP requires human rabies immune globulin plus one dose of vaccine on days 0, 3, 7, 14 and 28 (Essen regimen, recommendation World Health Organization [4]). In developing countries however, the vaccine is not always available and is expensive.

The WHO recommended gold standard of rabies diagnosis is the direct fluorescent antibody (DFA) test [4],[5]. In Chad, the DFA test was established in 2000 in the Laboratoire de Recherches Vétérinaires et Zootechniques de Farcha (LRVZ) [2]. It is currently the only location in the entire country where rabies diagnosis with the DFA test is possible. Suspected rabies is reported from most areas of the country [6]. The need for a fluorescence microscope, which is expensive and difficult to maintain, limits the overall use of the DFA test in developing countries.

At the Centers for Disease Control and Prevention (CDC), USA, a direct rapid immunohistochemical test (dRIT) has been developed to detect rabies virus using an immunoperoxidase technique [7]. The dRIT uses highly concentrated and purified biotinylated anti-nucleocapsid monoclonal antibodies to rabies virus. After incubation with a streptavidin-peroxidase complex, the antibody reagent is made visible with 3-amino-9-ethylcarbazole. The result can be read after less than one hour. Unlike the DFA test, the slides can be read using a light microscope. The dRIT recognizes all representative lyssaviruses examined to date. Using the test in the Serengeti, Tanzania, Lembo et al. found 100% sensitivity and specificity of the dRIT compared to the gold standard DFA test [8]. The preservation of rabies samples with glycerol saline (50% glycerol solution in 0.01 M phosphate-buffered saline), as it was used in the mentioned study [8], is an alternative to storage at −20°C in case of no availability of a freezer and may be used for current rabies diagnostic tests [9].

The objectives of the present study were 1) to evaluate the dRIT with samples from suspect urban domestic animals in N'Djaména, Chad, by comparing the results of the dRIT and the gold standard 2) to characterize the rabies viruses by genetic sequencing and 3) to re-estimate the incidence of canine rabies in N'Djaména.

## Materials and Methods

### Study place

The study location was N'Djaména, the capital of Chad, with a human population of 775,876 in 2001 [10]. The dog population of N'Djaména is estimated to be 23,575 (95% C.I. 14′579–37′921) [10]. The density of the dog population varies in different areas, depending on socio-cultural features of the inhabitants. The LRVZ is located in the western border of N'Djaména, 15 km away. This study was authorized by the National Veterinary Laboratory in Chad.

### Data collection and sample size

In the starting phase of the study all district offices, health centres and drug stores were informed about the project and posters were handed out describing the measures to be followed after an animal bite. Communities were motivated to bring suspected rabies cases (domestic animals) to LRVZ. Humans were voluntarily bringing in dogs for rabies diagnosis and cost of rabies diagnosis was charged to the applicant at US$ 9.80. For each applicant a questionnaire was filled out in French (Figure S1). These questionnaires also were part of routine data collection at LRVZ out of the study. The interview was held in French, Arabic or a Ngambay translation [2]. Data for the applicant (name and address), the suspicious animal (species, sex, rabies vaccination status, clinical signs, feral or not) and the bitten persons (age, relation to the animal) were recorded. An example of the questionnaire is presented in Figure S1. The availability of PEP was always guaranteed by holding a stock of human anti-rabies vaccine in case of a shortage of the vaccine in the city. Between September 2005 to November 2006, 48 fresh samples were collected, where in one case the absence of brain tissue precluded adequate diagnosis (Table 1).

**Table 1:** Number of samples tested by different methods

		DFA	dRIT	PCR
**Diagnosis of fresh samples at LRVZ**		47^a^	35	0
**Evaluation of dRIT**	retesting at LRVZ, Chad	68^b^	68^b^	0
	tested twice	10	10	
	tested three times	1	1	
	retesting at CDC, Atlanta	74^b^,^c^	74^b^,^c^	75^b^
	tested twice	8	10	
	tested three times	1		
**Total number of tests conducted**		209	198	75

a In one case of the collected 48 samples, the absence of brain tissue precluded adequate diagnosis.

b Including the 47 samples collected in this study and 21 and 28, respectively of archived samples obtained in previous investigations

c In one case of the 75 shipped samples, the remaining material was insufficient to perform the diagnostic tests.

### Diagnosis of rabies at LRVZ and storage of the samples

**Figure 1:**
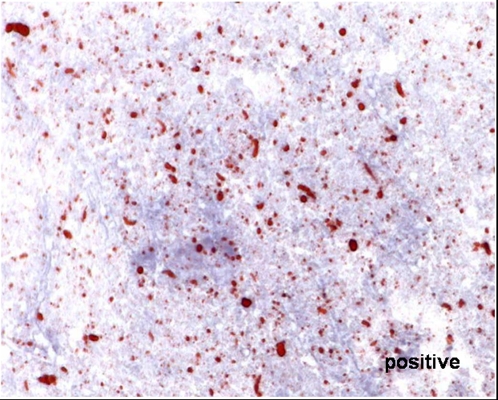
Picture of a positive result by the dRIT of a Chadian rabies sample, Magnification 400×.

**Figure 2:**
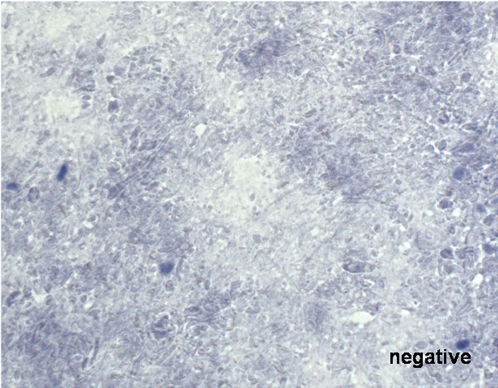
Picture of a negative result by the dRIT of a Chadian rabies sample, Magnification 400×.

To diagnose rabies the DFA test was used as a standard diagnostic tool [4]. The method is described elsewhere [5] and has an overall sensitivity of 99% and a specificity of 99.5% (Swiss rabies centre, unpublished data). For staining, the polyclonal SAD-RNP/FITC conjugate and the FITC Anti-Rabies Monoclonal Globulin were used. The dRIT was established in Chad after the training at the CDC of one of the study performer and the equipment and kit of the test were provided by CDC. The dRIT is described by Lembo et al. [8]. Positive results show red inclusions within bluish cell bodies (see Figure 1), which are not visible in negative samples (see Figure 2). The examination took place on the same day or, if the applicant delivered the animal at the end of the work time, the examination took place the next day. In fresh samples, where the dRIT was used parallel to the DFA (n = 35), the samples were read separately by two independent persons (laboratory technicians and veterinarians). Where available brain stem (medulla), cerebellum or Ammon's horn were used for the diagnosis. When these parts where not identifiable because the brains were decomposed, most fresh brain parts are chosen. On the day of the diagnosis, the same parts of brain as used for diagnosis were stored in two different manners at −20 degrees Celsius: in a 50% glycerol solution in 0.01 M phosphate-buffered saline, and without any additive in Petri dishes. Samples stored in glycerol solution were shipped to the CDC in Atlanta for further testing.

### Evaluation of the dRIT at the LRVZ and CDC

To evaluate the dRIT under different laboratory conditions, we retested the collected samples from the year 2005 up to the end of the study at the LRVZ and CDC by both, DFA and dRIT. All samples were stored without preservative at −20°C for a maximum of 10 months. In total 79 slides from 68 independent samples (including the 47 samples collected in this study and 21 of archived samples obtained in previous investigations) were read in Chad by both methods, where 10 samples were read twice and 1 sample three times (Table 1). 75 samples from the year 2005 up to the end of the study period (including the 47 samples collected in this study and 28 of archived samples obtained in previous investigations) were shipped to the CDC, stored in 50% glycerol solution. Before retesting at CDC they were stored in glycerol for three to 18 months. 83 and 84 slides from the 74 usable samples (in one case the remaining material was insufficient to perform diagnostic tests) were retested by the DFA and dRIT, respectively (Table 1). The CDC's standard protocol was used for the DFA [11]. For each sample, the condition was recorded as good, fair or poor, respectively. Samples in good condition were fresh with white brain tissue; in fair condition the brain structure was again recognizable but no longer fresh; poor-condition samples were putrid or dry, strongly smelling and the brain structure could not be recognized.

At the LRVZ, eight to eighteen slides per working day were prepared, stained and read by dRIT and DFA. All samples used were stored in Petri dishes without additives at −20°C. Afterward the samples were coded by one person, two other persons read the slides, independently and blinded to results. The comparison of the results read from the two persons and the known rabies status of each sample was performed after reading.

All samples tested at CDC were stored in 50% glycerol solution. Before testing, the glycerol was removed, and samples were washed twice by 10 min in phosphate-buffered saline. After washing, slide impressions for dRIT and DFA were made in the same day. One (dRIT) and two (DFA) days later, the slides were stained and read by both methods by one person. After reading, the results were compared with the rabies status of the sample. All samples which did not show a clear positive or negative result and those with contradictive results compared with the initial test of fresh sample in Chad were, after new coding, blindly retested by two different persons with the dRIT and DFA, respectively. One sample was tested a third time by the DFA to verify the result.

All involved persons were seasoned laboratory technicians or veterinarians.

### Molecular methods at CDC

The reverse-transcription polymerase chain reaction (PCR) was performed for all 75 samples shipped to the CDC. Total RNA was extracted from infected material (either original host brain or mouse brain following limited passages), using TRIzol™ (GIBCO-BRL Inc., Gaithersburg, MD, USA) according to the manufacturer's recommendations. The RT-PCR was performed as described elsewhere [12] with primers for amplification of the entire N gene. All negative PCR products were subjected to a nested reaction. The reaction is performed in the same way as the primary PCR with two µl of the primary PCR product used as a template and the primers were replaced by a pair of nested primers, bordering a fragment of 350 nucleotides within the frame amplified at the first step. Parameters of the nested reaction were the same those parameters of the primary PCR. All positive PCR products were purified and subjected to direct sequencing using the ABI Prism™ 377 DNA Sequencer (Applied Biosystems, Foster City, CA, USA) as described elsewhere [12].

Primary assembly, alignment, consensus generation and DNA translation were performed using BioEdit [13]. Neighbour joining (NJ) phylogenetic analysis was performed using Kimura-2-parameter in MEGA, version 2.1 [14]. Bootstrap support was estimated for 1000 replicates. Bootstrap values 70 and more were considered significant.

### Data analysis

The data were double entered in the Microsoft access, validated by Epi Info version 3.3.2. and analysed with SAS 9.1. The simple Cohen's kappa value was used for statistical comparison of the diagnostic tests (SAS command proc freq; table/agree). The confidence interval of rabies incidence is calculated by assuming a binomial distribution.

## Results

### Characteristics of suspicious animals and bitten persons

We received 48 suspicious animals in the period of September 2005 and November 2006. All suspicious animals brought to the laboratory were dead. Of these, 42 were positive and 5 negative by the DFA. In one case, the absence of brain tissue precluded adequate diagnosis. The characteristics of the suspicious animals and their diagnostic test result are presented in Table 2. A total of 90 people were bitten by the 42 rabid dogs, with 34 children under 12 years. All rabid animals had bitten at least one person. During the study period, we found three dogs with a negative result on both the DFA and dRIT in animals that had previously bitten one person each. However, despite the negative result, we recommended PEP to the exposed individuals.

**Table 2:** Characteristics of the 47 rabies suspicious animals

rabies diagnosis (based on DFA)		positive	negative
		mean (%)	mean (%)
total number		42 (89)	5 (11)
**species**	dog	41 (97.6)	3 (60.0)
	cat	1 (2.4)	1 (20.0)
	bat	0 (0.0)	1 (20.0)
**sex**	male	25 (60.5)	1 (20.0)
	female	9 (21.4)	0 (0.0)
	unknown	8 (19.1)	4 (80.0)
**ownership**	owned	30 (71.4)	3 (60.0)
	ownerless	11 (26.2)	2 (40.0)
	unknown	1 (2.4)	0 (0.0)
**manner of death**	killed	25 (60.0)	3 (60.0)
	died without intervention	7 (16.7)	2 (40.0)
	unknown	10 (23.8)	0 (0.0)
**rabies vaccination**	yes	1 (2.4)	0 (0.0)
	no	26 (61.9)	3 (60.0)
	unknown	15 (35.7)	2 (40.0)
**clinical signs**	anorexia	13 (31.0)	0 (0.0)
	aggressive	15 (35.7)	2 (40.0)
	change of behaviour	7 (16.7)	1 (20.0)
	weak, quite	4 (9.5)	0 (0.0)
	bitten for defence	0 (0.0)	1 (20.0)
	no sings reported	2 (4.8)	1 (20.0)

### Canine rabies incidence

During our study period between September 2005 and November 2006, the number of rabies positive dogs diagnosed at LRVZ was 42. Based on data from Mindekem et al [10], the dog population of N'Djaména was at 23′560 dogs in 2001. Assuming a stable dog/human proportion of 0.03 [10] and a growth rate of 2.5% of the human population [15] we estimated a dog population of 24′526 dogs in N'Djaména in 2006. Hence the annual canine rabies incidence is estimated to 1.71/1000 dogs per year (95% C.I. 1.45–1.98).

### Evaluation of the dRIT

Of the 47 received suspicious animals where diagnosis was possible, 35 were tested as fresh samples parallel by dRIT and DFA on the day we received the samples in the LRVZ (Table 1). 33 of these were positive and two negative. 12 samples were only tested by the DFA due to the absence of one study performer. We found a 100% agreement between the results of the two tests in fresh samples by both readers (Table 3). The simple Cohen's kappa coefficients between the rabies diagnosis as fresh sample and the retesting at LRVZ and CDC, respectively, are presented in the Tables 4 and 5. The coefficients are calculated overall and depending on the different sample's condition where we found higher kappa values in better sample conditions. In case of 64 samples the results of all retests (both, DFA and dRIT retests) correspond with the diagnosis as the fresh sample (based on DFA in Chad). After repeated reading (up to 3 times) of some of the samples, no indeterminate result remained.

**Table 3:** Results of DFA and dRIT by testing 35 fresh samples

	DFA		
dRIT	pos	Neg	total
pos	33	0	33
neg	0	2	2
total	33	2	35

**Table 4:** Retesting of dRIT and DFA in Chad: Simple Cohen's kappa coefficients calculated by comparing the retesting results with the DFA result of the samples in fresh condition

		DFA	dRIT	n
condition of the samples	overall^a^	0.70 (0.48–0.92)^b^	0.66 (0.44–0.88)	68
	good	1 (n.a.)	1 (n.a.)	6
	fair	0.89 (0.69–1)	0.90 (0.69–1)	41
	poor^c^	0.48 (0.14–0.82)	0.39 (0.07–0.71)	21

Samples stored without additive.

a all conditions taken together

b values in parentheses are 95% confidence intervals

c putrid or dry

**Table 5:** Retesting of dRIT and DFA at CDC: Simple Cohen's kappa coefficients calculated by comparing the retesting results with the DFA result of the samples in fresh condition

		DFA	dRIT	n
condition of the samples	overall^a^	0.60 (0.38–0.83)^b^	0.74 (0.54–0.95)	74
	good	0.87 (0.63–1.00)	1 (1.00–1.00)	38
	fair	0.59 (0.25–0.93)	0.76 (0.43–1.00)	29
	poor^c^	0.13 (−0.15–0.40)	0.22 (−0.20–0.64)	7

Samples stored in 50% glycerol

a all conditions taken together

b values in parentheses are 95% confidence intervals

c putrid or dry

Because of more ideal laboratory conditions and proper maintenance of the microscope, the slides were easier to read at CDC than at LRVZ.

### Results of molecular methods

In our study, 68 of the 75 samples tested positive, 7 negative by PCR. In all but one of the 10 cases where at least one of the retesting results by DFA or dRIT did not correspond to the rabies diagnosis as fresh sample, the PCR confirmed the fresh sample's results. In one case, the result of the PCR was negative, as well as the retesting result of DFA at CDC, but the results for the fresh sample as well as of the retesting result of dRIT were positive. The condition of this sample was poor (dry and hard). In 5 cases the PCR showed a positive result but all other tests negative. The opposite, positive results in all microscope tests but negative results in PCR were found twice.

**Figure 3:**
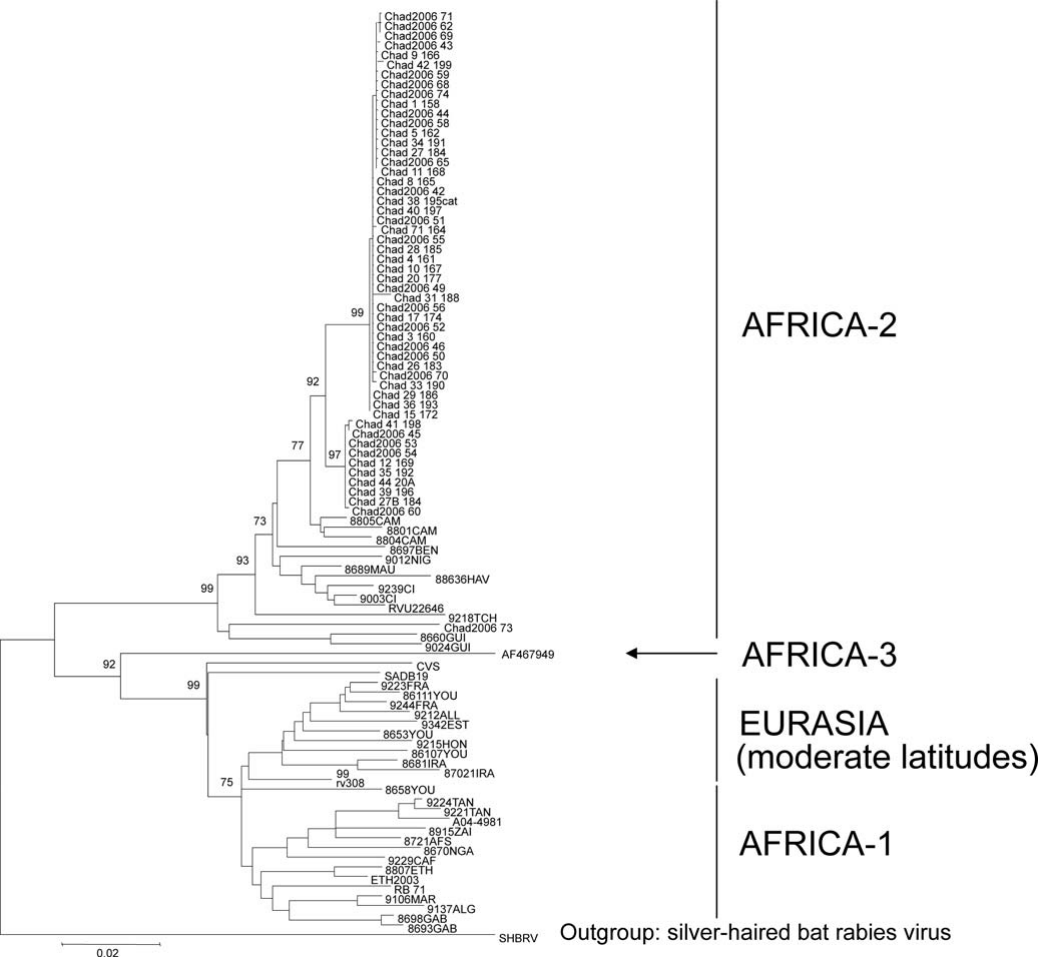
Phylogenetic tree of representative of all African groups (1, 2 and 3) after Kissi et al. (1995) and SHBRV as a root. Branch lengths correspond to differences between sequences (scale at the bottom of the figure). Only significant bootstraps values are shown. The samples of our study are labelled as Chad+number.

As a result, 53 complete and 15 limited N gene sequences were generated. Phylogenetically, all viruses were similar to each other and belonged to the African-2 group [16], circulating in dogs in different locations of Africa (Figure 3). The majority of sequences were subdivided into 2 clusters, different from each other in 18 nucleotides substitutions. Most of these substitutions were synonymous, with the only exception A/T_332_. Neither geographical nor host specificity was determined for these two clades due to the lack of geographic origin and host species of some of the samples. However, the vast majority of the samples are from dogs in N'Djaména. The sequences shared 98.1–100% nucleotide identity and 99.5–100% amino acid identity. The only exception was the isolate 200673 from Sidjie, which was more distant from these lineages, and shared with them 92.5–93.2% nucleotide identity. However, in this case most of the substitutions were synonymous as well. In addition, samples from the present study shared 91.0–94.3% nucleotide identity and 98.8–99.1% amino acid identity with virus 9218TCH, isolated in Chad in 1992 [16].

## Discussion

Rabies remains endemic in N'Djaména. The incidence, registered in our study, is similar to the previously communicated results [2]. The major vector is the dog (97.6% of all rabid animals). Rabies is highly underreported when estimations are conducted passively only (such as in this study), and based solely on public health laboratory submission data. For example, a study in Tanzania showed that the true occurrence is estimated 10 to 100 times higher by using recorded animal bite in health centres [17]. A similar study in N'Djaména should be done to assess the level of underreporting in Chad. Despite the information campaign at the start of the study and the motivation to bring suspected cases to the laboratory, the high percentage of 89% of positive cases in all suspicious samples indicates that only the suspected animals with typical signs, mainly aggressiveness and anorexia, were brought to the laboratory.

The dRIT is a simple diagnostic method. However, as with any test, the protocol has to be followed strictly and includes more steps than the DFA. As critical points for field use, the cold storage of the kit, and the use of various chemical reagents, have to be considered. However, we are able to obtain all secondary reagents for the test in N'Djaména. In fresh samples, the dRIT shows ideal results. Compared to the gold standard (DFA) the sensitivity and specificity are approaching 100%. Considering the retesting results at LRVZ and CDC on Table 4 and 5, respectively, we see that the condition of the sample influences the result of both tests, the dRIT and DFA. In Chad, we found kappa values of 1 in samples of good condition in the DFA and dRIT, but only 0.48 and 0.39 in samples of poor condition in the DFA and dRIT, respectively. At the CDC, the differences of kappa values between sample conditions are even higher: 0.87 (good condition) to 0.13 (poor condition) for the DFA and 1 (good condition) to 0.22 (poor condition) for the dRIT. We observed higher kappa values of the DFA as of the dRIT in Chad, but lower at CDC (Table 4 and 5). A main difference between the retesting studies at LRVZ and CDC was the storage of the sample: the samples had been stored in glycerol only before the CDC study. Glycerol saline is a convenient preservative in situations where freezing facilities are not promptly available [9]. Thus, the storage in glycerol seems to reduce the performance of DFA testing more than dRIT testing. Hence, dRIT has a potential to be used as a field diagnostic method, where sample freezing normally is not possible. Samples stored for up to 6 months without ever been frozen in 50% glycerol provide clearly positive results by dRIT after washing out the glycerol.

The diagnosis with molecular methods corresponded to all but ten of the results by dRIT and DFA. The sequencing results confirm our assumption that there is only a single major rabies virus variant in the dog population of N'Djaména, which has significant implication for control strategies.

Rapid diagnosis is the fundamental basis for surveillance and control of every major infectious disease. For an exposed person, rabies diagnosis is sensible if done immediately. In this case, the sample is still in reasonably fresh condition. To date, the dRIT has not exhibited any significant disadvantage compared to the DFA. Our study confirms the results of Lembo et al. who found a 100% sensitivity and specificity of the dRIT compared to the DFA by analysis of 159 samples of domestic and wild animals in Tanzania [8]. However, for a full validation of the dRIT, larger sample sizes are needed from a diversity of species, viruses, and working conditions.

A major advantage of the dRIT is that it utilizes a simple light microscope. The cost of a light microscope is more than ten times lower than for a fluorescent microscope, which is needed for DFA testing. Thus, the dRIT has a significant potential for diagnosing rabies in low income countries, and in field locations where rabies diagnosis is unavailable. To use this valuable new diagnostic test proper training and technology transfer is a priority.

## Supporting Information

Figure S1 Example of filled out questionnaire at LRVZ (part of the routine data collection of rabies diagnosis at LRVZ)
(0.12 MB TIF)

Alternative Language Abstract S1 Translation of the abstract into French by Jennifer Saurina
(0.03 MB DOC)

Alternative Language Abstract S2 Translation of the abstract into German by Salome Dürr
(0.03 MB DOC)

Checklist S1 STARD checklist
(0.03 MB DOC)
