# Supplementary figures and images for: Rabies Diagnosis for Developing Countries
Source: PLoS Negl Trop Dis. 2008 Mar 26;2(3):e206. doi: 10.1371/journal.pntd.0000206 (PMC2268742; doi:10.1371/journal.pntd.0000206)

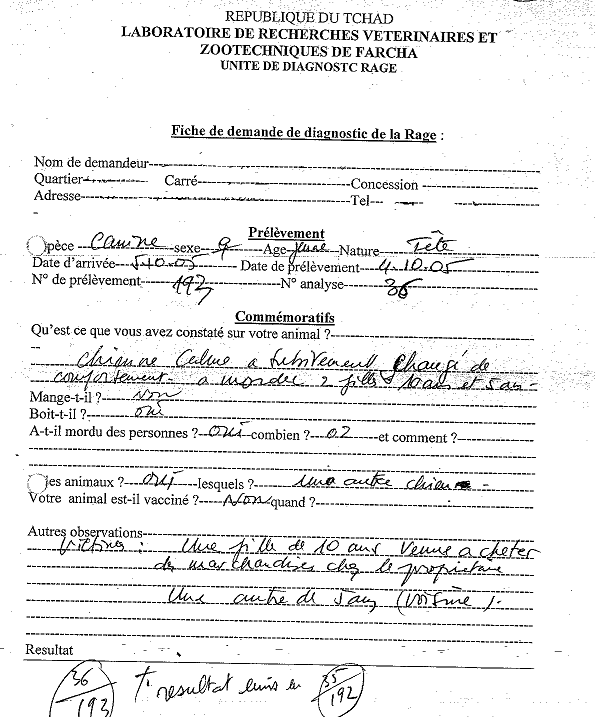

Supplement: Figure S1 — Example of filled out questionnaire at LRVZ (part of the routine data collection of rabies diagnosis at LRVZ) (0.12 MB TIF) [file pntd.0000206.s001.tif]
