# Supplementary material for: Rabies Diagnosis for Developing Countries
Source: PLoS Negl Trop Dis. 2008 Mar 26;2(3):e206. doi: 10.1371/journal.pntd.0000206 (PMC2268742; doi:10.1371/journal.pntd.0000206)
Supplement: Alternative Language Abstract S1 — Translation of the abstract into French by Jennifer Saurina (0.03 MB DOC) [file pntd.0000206.s002.doc]

### Résumé

**Introduction:** La rage est une maladie négligée. À travers le monde, elle cause 55'000 morts humaines par année, dont 99% sont dues à des expositions à des morsures canines. A N’Djaména, la capitale du Tchad, la rage est endémique avec une incidence de 1.71/1000 chiens (95% I.C. 1.45-1.98). L’étalon-or du dépistage de la rage est l’immunofluorescence directe (DFA test), qui requiert un microscope à fluorescence. Le CDC a développé un test histochimique meilleur marché, le test rapide immuno-histochimique (dRIT), puisqu’il ne nécessite qu’un microscope optique.

**Méthode/résultats** : Nous avons évalué le dRIT en examinant en parallèle 35 échantillons frais à l’aide des 2 méthodes, le DFA et le dRIT, au Laboratoire de Recherches Zootechniques et Vétérinaire (LRVZ) à N’Djaména, Tchad. La qualité de l’évaluation a été améliorée en réitérant l’examen sur les échantillons stockés (n=68 au Tchad, n=74 au CDC) à l’aide de DFA et dRIT. Les échantillons provenaient de chiens et de chats et dans un cas d’une chauve-souris. Lors de la comparaison avec le DFA, de très bons résultats ont été obtenus avec le dRIT. Nous avons trouvé 100% de concordance entre les résultats du dRIT et du DFA dans les échantillons frais (n=35). Les résultats du testage répété au CDC et au Tchad sont dépendants des conditions de conservation des échantillons. Lorsque l’échantillon était en bon état (tissu cérébral frais), nous avons trouvé un coefficient Kappa de Cohen de 0.87 (95% C.I. 0.63-1) à 1 entre les résultats du dRIT et du DFA. Dans les cas où la qualité des échantillons était médiocre, les valeurs de Kappa se trouvent entre 0.13 (95% C.I. -0.15-0.40) et 0.48 (95% C.I. 0.14-0.82). Pour les échantillons conservés dans le glycérol, les résultats du dRIT avaient plus tendance à s’accorder avec les résultats du test DFA des échantillons frais que des échantillons réexaminés.

**Conclusion/signification**: Le dRIT est une méthode de dépistage aussi fiable que l’étalon-or (DFA) dans les tissus frais. Il a l’avantage de ne requérir qu’un microscope optique, qui est 10 fois moins onéreux que le microscope à fluorescence. Les coûts réduits du dRIT suggèrent un grand potentiel pour rendre le diagnostic de la rage accessible à de larges populations. Grâce à cela, de nombreuses villes et régions rurales d’Afrique pourront contribuer à la lutte contre la rage en acquérant la capacité de dépister cette maladie.

**Mots clés** : rage canine, diagnostique, test immunohistochimique, N’Djaména, Tchad
