# Supplementary material for: Rabies Diagnosis for Developing Countries
Source: PLoS Negl Trop Dis. 2008 Mar 26;2(3):e206. doi: 10.1371/journal.pntd.0000206 (PMC2268742; doi:10.1371/journal.pntd.0000206)
Supplement: Alternative Language Abstract S2 — Translation of the abstract into German by Salome Dürr (0.03 MB DOC) [file pntd.0000206.s003.doc]

### Zusammenfassung

**Hintergrund:** Kanine Tollwut ist eine in ihrer Bedeutung unterschätzte Krankheit, welcher weltweit 55’000 Menschen pro Jahr zum Opfer fallen. In 99% aller Fälle ist der Hund für die Übertragung des Virus auf den Menschen verantwortlich. Die Inzidenz der Hundetollwut in N’Djaména, der Hauptstadt des Tschads, liegt bei 1.71/1000 Hunden (95% C.I. 1.45–1.98). Als Standardmethode für die Tollwutdiagnostik wird heute der Immunfluoreszenz-Test (DFA) verwendet, welcher ein Fluoreszenzmikroskop voraussetzt. Im CDC wurde ein histochemischer Test, der direct rapid immunohistochemical test (dRIT) entwickelt, welcher auf einem kostengünstigeren Lichtmikroskop basiert.

**Methoden/wichtigste Resultate:** Wir evaluierten den dRIT im staatlichen Veterinärlabor in N’Djaména, indem wir 35 frische Tollwut-Verdachtsfälle mit dem DFA und dem dRIT untersuchten. Zusätzlich wurden gelagerte Verdachtsfälle mittels DFA und dRIT getestet (n=68 in N’Djaména und n=74 im CDC), um die Power der Evaluation zu erhöhen. Sämtliche Proben stammten von Hunden oder Katzen, in einem Fall von einer Fledermaus. Der dRIT zeigte ausgezeichnete Resultate. Wir errechneten eine 100%ige Übereinstimmung zwischen dem dRIT und dem Gold Standard DFA in frischen Proben (n=35). Beim Testen der gelagerten Proben hängt das Resultat vom Zustand der Probe ab. Für Proben in gutem Zustand wurde ein Cohen’s Kappa-Wert bezüglich dem DFA-Testresultat als frische Probe von 0.87 (95% C.I. 0.63–1) bis 1 errechnet. Für Proben in schlechtem Zustand liegen die Kappa-Werte zwischen 0.13 (95% C.I. -0.15–0.40) und 0.48 (95% C.I. 0.14–0.82). Bei Proben, die in Glycerol gelagert wurden, zeigten die dRIT- Resultate bessere Werte in Bezug auf das Testresultat als frische Probe als die DFA-Resultate.

**Konklusion/Bedeutung:** Der dRIT ist in frischen Proben ein ebenso starker Test wie der heutige Gold Standard. Sein grosser Vorteil ist die Verwendung eines normalen Lichtmikroskopes, welches 10 Mal günstiger ist als ein Fluoreszenzmikroskop. Reduzierte Kosten bedeuten wiederum eine erhöhte Möglichkeit, den Test in städtischen und ländlichen Labors zu etablieren, in denen zurzeit keine Diagnostik möglich ist, um der von Tollwut betroffene Populationen in Afrika den Zugang zur Tollwuterfassung und –Bekämpfung zu erleichtern.

**Schlüsselwörter:** Kanine Tollwut, Diagnostik, immunohistochemischer Test, N’Djaména, Tschad
