# Supplementary material for: Rabies Diagnosis for Developing Countries
Source: PLoS Negl Trop Dis. 2008 Mar 26;2(3):e206. doi: 10.1371/journal.pntd.0000206 (PMC2268742; doi:10.1371/journal.pntd.0000206)
Supplement: Checklist S1 — STARD checklist (0.03 MB DOC) [file pntd.0000206.s004.doc]

### STARD checklist for the study: “Rabies Diagnosis for Developing Countries”, submitted at PLoS NTDs

Item 1:

An open title was chosen for this paper.

Item 2:

On page # 5, described by the first aim of the study.

Item 3:

Study population: domestic animals brought to the Chadian National Veterinary Laboratory. Location of the samples: Mostly from N’Djaména, but there was no exclusion criteria. On page # 5.

Item 4:

The recruitment was done passively (communities were motivated to bring suspected rabies cases (domestic animals) to LRVZ.). Applicants brought the animals to the laboratory when rabies was suspected (shown signs of the animal varied). On page # 5.

Item 5:

The study population was a consecutive series of participants defined by item 3 and 4.

Item 6:

The data collection was planned before the performance of the index test and reference test. As described on page # 6, samples were retested after the first performance of the tests, too.

Item 7:

The reference standard test is the DFA (direct fluorescent antibody test). For the rational we cite a reference on page # 6.

Item 8:

The index test is shortly described on page # 4 (including picture and reference). On page # 6 we cite a previous study concerning the index test. For the rational of the reference test we cite a reference on page # 6.

Item 9:

The positive and negative result are illustrated in Figure 1 (page # 5).

Item 10:

Described on page # 6 and 7.

Item 11:

Described on page # 7.

Item 12:

Described on page # 8 (data analysis).

Item 13:

Reproducibility of the test was shown by the two retesting sessions at LRVZ and CDC (Table 4 and 5).

Item 14:

First sentence of the result part (page # 8).

Item 15:

Table 2, description on page # 8.

Item 16:

Described at the beginning of the different result parts, on page 8, 9 and 10, respectively. Sample sizes are presented in Table 1.

Item 17:

The two tests were done parallel or with one day difference, as described on page # 7 and 9.

Item 18:

Symptoms of the disease are described in Table 2, separated into positive and negative cases.

Item 19:

Table 3.

Item 20:

Because of more ideal laboratory conditions and proper maintenance of the microscope, the slides were easier to read at CDC than at LRVZ (page # 10).

Item 21:

Table 4 and 5.

Item 22:

Described on page # 10.

Item 23:

Description of the dependency of the results from the samples condition and whether it was stored in glycerol or not. Showed in Table 4 and 5 and discussed in the discussion part (page # 11 and 12).

Item 24:

See item 13. In the discussion it is mentioned that further evaluations are needed with bigger sample sizes.

Item 25:

Discussed in the last part on the discussion (on page 12).
